# Helicobacter Pylori-Induced Gastric Infections: From Pathogenesis to Novel Therapeutic Approaches Using Silver Nanoparticles

**DOI:** 10.3390/pharmaceutics14071463

**Published:** 2022-07-14

**Authors:** Romelia Pop, Alexandru-Flaviu Tăbăran, Andrei Paul Ungur, Andrada Negoescu, Cornel Cătoi

**Affiliations:** Department of Anatomic Pathology, Faculty of Veterinary Medicine, University of Agricultural Sciences and Veterinary Medicine, 400372 Cluj-Napoca, Romania; romelia.pop@usamvcluj.ro (R.P.); andrei.ungur@usamvcluj.ro (A.P.U.); andradanegoescu@yahoo.com (A.N.); cornel.catoi@usamvcluj.ro (C.C.)

**Keywords:** silver nanoparticles, *Helicobacter pylori*, infection, treatment, antibacterial, pathogenesis, antibiotic resistance

## Abstract

*Helicobacter pylori* is the first formally recognized bacterial carcinogen and the most important single digestive pathogen responsible for the induction of gastroduodenal diseases such as gastritis, peptic ulcer, and, finally, gastric neoplasia. The recently reported high rates of antimicrobial drug resistance hamper the current therapies of *H. pylori*, with therapeutic failure reaching up to 40% of patients. In this context, new treatment options and strategies are urgently needed, but the successful development of these new therapeutic tools is conditioned by the understanding of the high adaptability of *H. pylori* to the gastric acidic environment and the complex pathogenic mechanism. Due to several advantages, including good antibacterial efficiency, possible targeted delivery, and long tissular persistence, silver nanoparticles (AgNPs) offer the opportunity of exploring new strategies to improve the *H. pylori* therapy. A new paradigm in the therapy of *H. pylori* gastric infections using AgNPs has the potential to overcome the current medical limitations imposed by the *H. pylori* drug resistance, which is reported for most of the current organic antibiotics employed in the classical therapies. This manuscript provides an extensive overview of the pathology of *H. pylori*-induced gastritis, gastric cancer, and extradigestive diseases and highlights the possible benefits and limitations of employing AgNPs in the therapeutic strategies against *H. pylori* infections.

## 1. Introduction

*Helicobacter pylori*, a spiral-shaped or coccoid [1] Gram-negative bacterium is the most common infectious agent of gastric diseases worldwide. *H. pylori* infect 30–70% of the world’s population, being one of the leading causes of gastrointestinal diseases, producing functional dyspepsia, acute and chronic gastritis, and peptic ulcer in up to 20% of infected cases. Importantly, following *H. pylori* infection, there is a 3–6 times higher risk of developing aggressive gastric cancers, including gastric adenocarcinoma and mucosa-associated lymphoid lymphoma (MALT lymphoma) [2]. In this context, *H. pylori* is the single bacterium classified as a group I carcinogen by the World Health Organization (WHO) [3]. In this context, the eradication of *H. pylori* infection is indicated as the first choice of treatment for several of these gastric malignancies, including MALT lymphoma [4]. In addition to the well-established gastric pathologies, recently, a considerable amount of evidence has linked *H. pylori* infection with several extragastric diseases within the nervous, cardiovascular, and immune systems [5], such as dementia, Alzheimer’s disease [6], Parkinson’s disease [7], Guillain–Barré syndrome [8], iron deficiency anemia/aregenerative anemia [9], coronary atherosclerosis [10], and nonalcoholic steatosis [11]. The *H. pylori* infection is typically acquired in early childhood, either by fecal–oral or oral–oral route, and in the absence of effective treatment, it lasts a lifetime [12,13]. This indicates a strong adaptation to the biological niche represented by the mucous layer covering the gastric epithelial cells. The resistance mechanism of *H. pylori* within the low-pH environment of the stomach is particularly complex, involves the expression of several genes considered key pathogenic factors (as CagA, VacA, BabA, urease, etc.), and determines several particularities in the currently employed therapies, such as simultaneous administration of several active molecules (typically at least two antibiotics and a proton pump inhibitor) and long-term treatments (lasting at least 2 weeks) [14].

### 1.1. Current Treatment Protocols for H. pylori Infection and Emerging Antibiotic-Resistant Strains

The first-line option for *H. pylori* infection is represented by a standard triple therapy (STT) consisting of two antibiotics, clarithromycin and amoxicillin or metronidazole in combination with a proton pump inhibitor (PPI). The second-line option for *H. pylori* treatment is represented by bismuth-based quadruple therapy. If the first- and second-line therapy fails, triple therapies (based on levofloxacin and/or rifabutin in combination with amoxicillin) can be an option. Furthermore, sequential therapy including initially dual administration of a proton pump inhibitor (PPI) plus amoxicillin followed by a triple therapy including a PPI, clarithromycin, and tinidazole was developed as a therapeutic alternative [15].

Successful treatment of *H. pylori*-induced gastritis has become a challenge in recent years [16] due to several factors, including the emergence of new antibiotic-resistant strains (e.g., up to 72.5%-resistant for amoxicillin, 58.8% for clarithromycin, and 34.9% for metronidazole) [17,18] and poor patient compliance. In the “antibiotic resistance era”, the low eradication rate and suboptimal results of the classical therapy are reported in both children and adults, with some studies showing therapeutic failure in up to 40% of the children and 38% of the adults subjected to the standard triple therapy for *H. pylori* infections in China [19,20]. Bismuth-based quadruple therapy has been proven to have better results regarding the treatment of *H. pylori.* Luminal bismuth is responsible for the action of bismuth salts within the upper gastrointestinal system. Inhibiting a variety of enzymes, ATP generation, and the bacteria’s adhesion to the stomach mucosa are only a few of the ways that bismuth has a direct bactericidal effect on *H. pylori*. Additionally, bismuth promotes the release of prostaglandin, epidermal growth factor, and bicarbonate, which are mucosa-protective substances that aid in the healing of ulcers. No reports of bismuth resistance exist as of yet. The suboptimal results in the classical therapies merged with the need for the development of new treatment agents for *H. pylori*-induced gastritis [19]. Based on the high rates of antibiotic resistance and recent epidemiological evidence, in 2017, the WHO listed *H. pylori* among the sixteen antibiotic-resistant bacteria ranked as high-priority bacteria that pose the greatest threat to human health worldwide [21].

### 1.2. Potential Use of AgNPs as a Complementary or Alternative Therapy for H. pylori in the Context of Antibiotic Resistance

Pathogens that are resistant to older antimicrobials and compounds that are regarded last-resort treatments have continued to emerge as a result of antibiotic use. Therapy effectiveness is significantly harmed by antimicrobial resistance, and the risk of cross-infection in hospitals has been on the rise. Resistance causes ineffective empirical therapy, a delay in beginning treatment, and the use of less effective, more harmful, and more expensive medications [22,23,24].

Growing rates of antibiotic resistance of *H. pylori* are reported worldwide. This is highlighted by the overall low resistance rates reported in the first decade of 2000 [25]. In response to this high rate of resistance to antibiotics used in the classical therapy, recently, the World Health Organization (WHO) designated the clarithromycin-resistant strains of *H. pylori* as a high priority for antibiotic research and the development of alternative solutions. A study conducted from April 2008 to June 2009 in 18 European countries showed that rates of resistance for adults were 34.9% for metronidazole, 14.1% for levofloxacin, and 17.5% for clarithromycin [18]. In the period from 2008 to 2017, there was another study conducted by Megruad et al. [26] in the same 18 European states that showed the resistance rates were 38.9% for metronidazole, 15.8% for levofloxacin, and 21.4% for clarithromycin.

Antibiotic resistance has increased worldwide, which seriously hampers the eradication rate of the frequent chronic infection. Areas covered: *H. pylori* MDR rates are discussed, mostly in the recent articles published since 2015. Present approaches and future directions to counteract MDR are outlined. Some studies have shown that probiotics have an inhibitory effect on *H. pylori* but other studies have proven that their effect is limited [27,28]. Approximately 20% of patients do not respond positively to the recommended treatment regimen. Inadequate patient compliance, obesity, smoking, reinfection, genetic polymorphisms in CYP2C19, poor patient maintenance, improper alimentary regimen, antibiotic resistance, disease entities associated with *H. pylori* infection, antibiotic degradation by the acidic stomach environment, and pharmacological activity of the prescribed drugs are the major causes of treatment failure [3,29,30]. The main cause of eradication failure is represented by the resistance to antibiotics [31].

Because of the major need to avoid antibiotic resistance and the high infection rate of *H. pylori*, it is necessary to develop an effective alternative to the typical antibiotic therapy. The recent studies have shown an efficient antimicrobial effect of metal nanoparticles using their potential in the medical field [32,33,34]. It is known that metallic nanoparticles were synthesized centuries ago because of their intrinsic physical and chemical characteristics, especially their optoelectronic properties and surface plasma resonance [35]. Silver-based nanoparticles have been proven to be effective against multiple microorganisms, such as bacteria, viruses, and bacterial biofilm. Due to the attachment on the cell surface, silver nanoparticles interact with bacteria, changing the wall structure and causing damage to cell functions, increasing their permeability by pore formation and affecting the respiratory enzymes. These actions eventually lead to cell death [36]. One of the major public health problems caused by the infection with multidrug-resistant bacteria is morbidity and mortality which cause the need to implement measures to control the infection.

The development of new antibacterial drugs against *H. pylori* and identification of new drug targets for the diseases caused by persistent *H. pylori* infections must first take into account the complex diseases with complicated pathogenesis and the high adaptability of *H. pylori* to the gastric environment. Next, we present briefly the main pathologies induced by persistent *H. pylori* gastric infections, highlighting the potential usage of AgNPs in the therapy and prevention of these infections in the context of pathogenicity.

In addition to the usage of nanoparticles as direct antibacterial agents, currently, the nanotechnology of advanced encapsulation strategies enables novel material–cell interactions, such as reversible encapsulation of living-single cells [37], offering fascinating biomedical opportunities in drug delivery and transplantation [38] Drug delivery systems are a relatively young but quickly developing technology. In these systems, therapeutically effective drugs or imaging molecules are delivered to the targets using nanoscale materials. These carrier systems, which improve medication formulation, targeting, and controlled release, are fundamental to personalized medicine and depend heavily on nanoparticles and nanostructured materials. Such systems can deliver medicines at a predetermined pace and in a preplanned manner to a specific region; as a result, the drug’s bioavailability is increased and its negative effects are decreased. Drugs may be placed onto nanoparticles during manufacturing or may be physically or chemically adsorbed into the surface of the particles using various adsorption techniques [39,40,41].

## 2. Methods

### 2.1. Literature Search

The literature search followed the Preferred Reporting Items for Systematic Reviews and Meta-Analyses (PRISMA) guidelines [42]. The data were systematically searched in the following databases: Web of Science (“All databases” selected), PubMed (“All databases” selected), and Google Academic (last accessed on 24 June 2022), using keywords “*Silver nanoparticles H. pylori*”, “*Ag nanoparticles H. pylori*”, “*Silver nanoparticles Helicobacter*”, “*Ag nanoparticles Helicobacter*”, “*Silver nanoparticles pylori*”, and ”*Ag nanoparticles pylori*”. The data were searched separately by two of the researchers (P.R. and T.A.F.).

Through the initial search procedure, 242 items in total were found: Web of Science—170 results (“*Silver nanoparticles H. pylori*”, *n* = 30; “*Ag nanoparticles H. pylori*”, *n* = 8; “Silver nanoparticles Helicobacter”, *n* = 50; “Ag nanoparticles Helicobacter”, *n* = 12; “*Silver nanoparticles pylori*”, *n* = 55; ”*Ag nanoparticles pylori*”, *n* = 15); PubMed—58 results (“*Silver nanoparticles H. pylori*”, *n* = 12; “*Ag nanoparticles H. pylori*”, *n* = 8; “*Silver nanoparticles Helicobacter*”, *n* = 11; “*Ag nanoparticles Helicobacter*”, *n* = 7; “*Silver nanoparticles pylori*”, *n* = 12; ”*Ag nanoparticles pylori*”, *n* = 8); Google Academic (only title were searched)—14 results were found (“*Silver nanoparticles H. pylori*”, *n* = 2; “*Ag nanoparticles H. pylori*”, *n* = 0; “*Silver nanoparticles Helicobacter*”, *n* = 12; “*Ag nanoparticles Helicobacter*”, *n* = 0; “*Silver nanoparticles pylori*”, *n* = 0; ”*Ag nanoparticles pylori*”, *n* = 0) (Supplementary File S1).

### 2.2. Selection of Studies

The specific search algorithm and results are provided as a PRISMA diagram (Supplementary File S1).

A total of 154 duplicates were excluded. Another 66 results were excluded because of other reasons: “*Helicobacter pylori*” was not found in the content (*n* = 13); “Silver nanoparticles” (*n* = 16) were not found in the content; both “*Helicobacter pylori*” and “Silver nanoparticles” were not found in the content (*n* = 35). Furthermore, review articles were excluded (*n* = 2). The remaining articles (*n* = 18) were included in the current study, and the data were further extracted and analyzed.

## 3. The Pathology of *Helicobacter pylori* Infection: From Inflammation to Gastric Neoplasia

**Figure 1:**
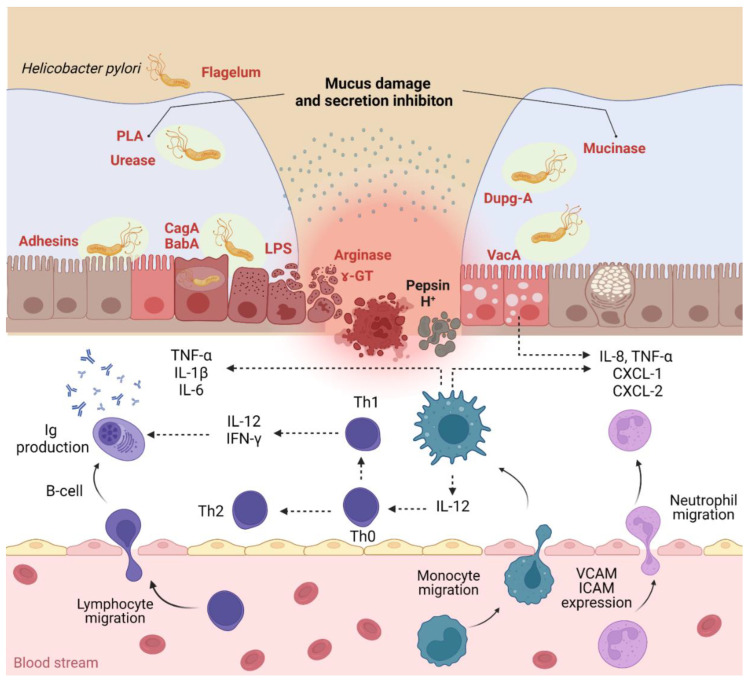
*H. pylori* infection pathogenicity and inflammatory response (PLA—phospholipases; Dupg—duodenal ulcer-promoting gene A; CagA—cytotoxin-associated gene A; BabA—blood group antigen-binding adhesin; VacA—vacuolating cytotoxin A; CXCL—chemokine ligand; VCAM—vascular cell adhesion molecules; ICAM—intercellular cell adhesion molecules). Created using BioRender (adapted from [48,49]).

The pathogenic process of *H. pylori* is particularly complex and consists of three important steps: (1) colonization, (2) immune escape, and (3) disease induction (Figure 1). The bacterium colonizes the stomach, settling in the deep portions of the gelatinous mucous layer lining the gastric mucosa and between the mucous layer and the apical surface of the epithelial cells of the gastric mucosa. The gastric mucosa is well-protected against bacterial infection. *H. pylori* is well-adapted to this ecological niche, having unique characteristics that allow it to enter mucus, attach to epithelial cells, evoke the immune response and, consequently, persistent colonization and transmission. The colonization of the mucosa is due to a set of aggressive enzymes (urease, mucinase, peptidases, etc.) that render it pathogenic, the bacterium being located under the mucous layer, between the epithelial cells, and around the gastric crypts. Bacterial phospholipases destroy the bilipid layer of the gastroduodenal epithelial cell membrane. Being a bacterium with very high mobility, it has flagella that allow it to easily cross the viscous layer of the mucosa to reach the levels of gastric mucosa cells where pH is almost neutral. Colonization of the mucosa would not be possible if the microbe did not protect itself from the acidic environment of the stomach. The next aggression factor is urease, a surface enzyme in the bacterial body which hydrolyzes urea, causing an increased amount of ammonia, which surrounds the bacterial body like a cloud. The ammonia formed is associated with hydrochloric acid forming extremely toxic products: hydroxylamine and monochloramine [43]. The following factors participate in the induction of gastroduodenal diseases: adaptation factors, bacterial survival in the host environment, and virulence factors (Table 1). Pathogenic factors can be exemplified by the protein products of CagA, VacA, IceA, and BabA genes [44].

Urease produced by *H. pylori* catalyzes the hydrolysis of urea into ammonia and carbon dioxide, which are necessary for gastric colonization and the protection of the bacillus from the effects of gastric acid. Hydroxide ions generated by the reaction of water with ammonia can contribute to damage to the gastric epithelial mucosa, and surface proteins are chemotactic for polymorphonuclear cells and monocytes. It also secretes platelet-activating factors, activates monocytes, produces superoxide, interleukin 1, tumor necrosis factor, protease, and phospholipases, which degrade the glycoprotein–lipid complex of the mucous layer. The first pathogenicity factor described was the CagA gene (cytotoxin-associated gene), which is part of the pathogenicity island (PAI) [45]. The protein secreted by this gene hitting the epithelial cells of the gastric mucosa induces their modification, damaging their cytoskeleton, and induces the synthesis of protein kinases, nuclear factor NF-kB, proinflammatory interleukins 1, 6, 8, chemotactic monocyte protein, tumor necrosis factor (TNF) [46]. Interleukin 8 is a powerful factor that accelerates the movement of neutrophils, and the chemotactic monocyte protein accelerates the movement of monocytes to the source, with the infiltration of the gastroduodenal mucosa with inflammatory cells: neutrophils and monocytes. This explains the predominance of intensely inflammatory reactions in people with pathogenic CagA+ strains compared to CagA strains. Some authors believe that CagA+ *H. pylori* strains inhibit phagocytosis, compromising the host’s immune response [47].

Patients infected with CagA+ *H. pylori* have a 12 times higher risk of developing intestinal metaplasia of the gastroduodenal mucosa, which explains the possibility of further development of gastric cancer [50]. Another pathogenicity factor is VacA (vacuolating cytotoxin gene) which secretes a cytotoxic protein that damages epithelial cells, forming pores in the cytoplasmic membrane, increasing their permeability to the formation of vacuoles inside the cell, so it is a gene encoding the cytotoxin inducing vacuolization [51]. The regeneration of the ulcer defect depends on the balance between the processes of proliferation and apoptosis. The chronic ulcerative defect is caused by numerous factors, including the high apoptotic activity of epithelial cells. The predominance of apoptosis over-proliferation can be the cause of gastroduodenal ulcers with persistent evolution and prolonged scarring. Conversely, the predominance of proliferative processes over apoptosis increases the number of epithelial cells whose nucleus contains damaged, immature DNA, and thus the accumulation of mutations, which explains the carcinogenic mechanism of *H. pylori*. In response to long-term *H. pylori* infection and permanent damage to the gastroduodenal mucosa, cell proliferation processes intensify [52]. Cytotoxic strains CagA and VacA are characterized by greater stimulation of the proliferation processes. It was found that after the eradication of *H. pylori* in the antral region, the proliferation processes are reduced considerably and early, while in the gastric body, the normalization of the imbalance of the proliferation processes takes place much later. Some authors have shown in vivo that CagA-positive *H. pylori* strains (CagA+ *H. pylori*) stimulate proliferation but, unlike *H. pylori*-negative strains (CagA– *H. pylori*), inhibit apoptosis, having a lower apoptotic index [53]. In 1998, R. Peek et al. [51] identified the IceA gene (induced by contact with the epithelium). This gene exists in two alleles, IceA1 and IceA2; the proteins secreted by this gene ensure adhesion to epithelial cells of the gastroduodenal mucosa. According to some authors, the presence of the IceA1 gene determines the development of gastric ulcers and the presence of neutrophil infiltration of the gastroduodenal mucosa [54]. Another factor in the adhesion of *H. pylori* to the gastroduodenal epithelium is the product of the BabA gene (blood group antigen-binding adhesin) which is a mediator of *H. pylori* adhesion to the Lewis antigen system, which ensures the persistence of the bacterium in the gastroduodenal area. The presence of this gene is considered a marker of the development of complications [53]. *H. pylori* cause a continuous inflammation of the gastric mucosa in all infected people. This inflammatory response initially consists of the recruitment of neutrophils, followed by T and B lymphocytes, plasma cells, and macrophages, with alteration of the epithelial layer (Figure 1). Differences in the immune response reflect the diversity of clinical manifestations of *H. pylori* infection [55]. *H. pylori* can live for years and sustain an intense inflammatory process because of their toxins such as vacuolating cytotoxin encoded by the VacA gene and immunogenic protein CagA encoded by the CagA gene (cytotoxin-associated gene, found only in the strains that produce an aggressive form of gastroduodenal diseases). The last one is located in the *H. pylori* pathogenicity island (PAI) [56,57,58]. To survive in a low-pH environment, *H. pylori* produce urease (the main virulence factor used for diagnosis), an enzyme that helps in the colonization of gastric mucosa. Urease has a major role in the neutralization of the acid fluid by catalyzing the degradation of urea to carbon dioxide and ammonia and raising the pH of the environment. It was showed that for the production of urease, *H. pylori* uses at least seven genes: ureEFGH encodes the accessor proteins that are in control of integrating nickel in the active center of the enzyme while the ureAB gene encodes two genes of the enzyme and ureI encodes the proteins that are in charge of urea transport into the target cell. All strains of *H. pylori* contain two phospholipases (A and C). The role of these two enzymes is to destroy the mucous layer [57].

**Table 1:** Synopsis of the main *H. pylori* virulence factors and their pathogenic mechanism [49,56,59,60].

Bacterial Element	Virulence Factors	Mechanism of Bacterial Virulence
**Flagella**	Flagellum	Bacterial motility
		Leukocyte chemotaxis
		Biofilm formation
		Inflammation and immune response
**Adhesins**	Blood group antigen-binding adhesin (BabA)	Adherence to gastric epithelial cells
		Toxins delivery
		Increasing inflammatory responses
	Sialic acid-binding adhesin (SabA)	Neutrophil activation
		Colonization
		Oxidative stress
	Adherence-associated lipoproteins A and B	Adherence to gastric epithelial cells
		Colonization
		Biofilm formation
		Release of proinflammatory factors
	LacdiNAc-specific adhesin (LabA)	Adherence to gastric epithelial cells
**Enzymes**	Urease	Protection from acidity
		Colonization
		Bacterial nutrition
		Control of the host immune response
		Platelet activation
		Angiogenesis
	Catalase	DNA damage and mutagenesis
		Induction of inflammation
		Survival of phagocytosis
	Superoxidase dismutase (SOD)	Gastric colonization
		Protection from reactive oxygen species
		Inhibition of the synthesis of cytokines
		Activation of macrophages
	Arginase	Apoptosis
		Protection from acidity
		Dysregulation of the immune response by inhibition of T and B cells production
		Macrophage apoptosis
	Phospholipases (PLAs)	Degradation of lipids
		Lysis of the mucous layer
		Chronic inflammation induction
		Gastric colonization
	Cholesteryl ꭤ-glucosyltransferase	Protection from immune responses and phagocytosis
		Secretion of proinflammatory factors
		Antibiotic resistance
		Bacterial growth
	ɤ-glutamyl-transpeptidase (GGT)	Stimulation of the release of SOR
		Apoptosis and necrosis
		Induction of the release of proinflammatory factors
		DNA damage
		Decrease in cell viability
	High-temperature-requirement serine protease A (HTRA)	Damage of the gastric epithelium
		Bacterial mobility
**Proteins**	Cytotoxin-associated gene A (CagA)	Induction of the inflammatory response
		Bacterial motility
		Activation of fibroblasts
		Oncogenesis (by dysregulation of the RUNX3, ASPP2, CDX1, and AFADIN genes)
		Decrease in microRNA-134, PDCD4, GSK-3
		Tumor progression by induction of cancer stem cell-like characteristics
	Vacuolating cytotoxin A (VacA)	Induction of autophagy and formation of autophagosomes
		Induction of cellular apoptosis and necrosis
		Dysregulation of the immune response by inhibition of T and B cells production
	Outer inflammatory protein A (OipA)	Induction of apoptosis
		Induction of the release of proinflammatory factors
		CagA delivery Stimulation of the microRNA-30b level
	Outer membrane protein Q (HopQ)	Bacterial adherence to gastric epithelial cells
		Protection from gastric acidity
		Induction of the release of proinflammatory factors
		Inhibition of the immune response
	Outer membrane protein Z	Adherence to gastric epithelial cells
		Increase in gastric secretion
	Neutrophil-activating protein (NAP)	Stimulation of neutrophils adhesion to gastric epithelial cells
	Heat shock proteins (Hsps)	Maintenance of intact functional and structural characteristics of cellular proteins
		Chronic inflammation and angiogenesis
		Adherence to gastric epithelial cells
		Induction of apoptosis autophagy
		Urease activation
		Gastric tumor cells migrations
**Toxins**	Lipopolysaccharide (LPS)	Stimulation of the inflammatory response by neutrophil activation
		Induction of bacterial protection
		Impairment of the gastric mucosa mucus production
**Others**	Lewis antigens	Protection from host defense
		Bacterial protection
		Adhesive proprieties
	Duodenal ulcer promoting gene A (DupA)	Increase in the inflammatory response
		Induction of apoptosis (intrinsic pathway)
		Bacterial resistance to the acidic microenvironment

There are several gastric and extragastric pathologic effects of the chronic *H. pylori* infection. To hypostatize which point of *H. pylori* infection would benefit from AgNP therapy, in the next section, the main gastric lesions (including inflammatory and neoplastic changes) and extragastric pathologies are detailed.

### 3.1. Gastric Inflammation Induced by H. pylori Infection

**Figure 2:**
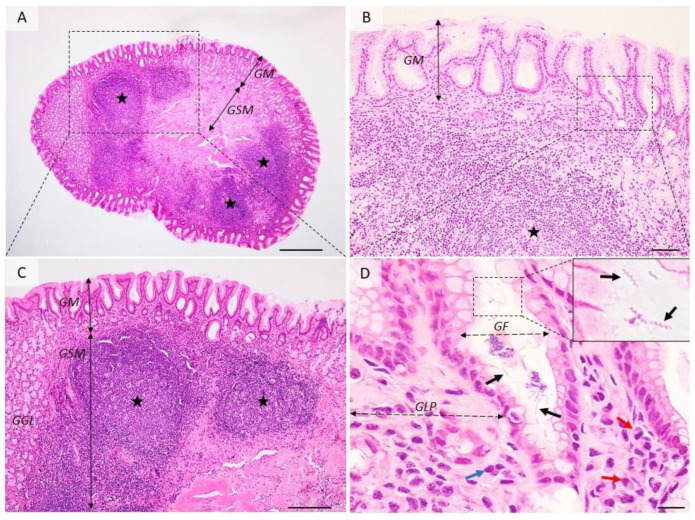
Histological aspects of follicular gastritis. (**A**–**C**) The lamina propria is multifocally distended and the gastric glands are displaced by many coalescing lymphoid follicles showing normal lymphocyte maturation. (**D**) Detailed demarcated area from (**B**). Within the gastric pits and superficial epithelium, there are many extracellular spiral-shaped bacteria measuring up to 7 µm. The lamina propria is infiltrated by many plasma cells (red arrow) and lymphocytes (blue arrow). GM = gastric mucosa; GSM = gastric submucosa; GGL = gastric glands; GF = gastric foveolar cells; GLP = gastric lamina propria; red arrows—plasma cells, blue arrow—fibroblasts, black arrows—*Helicobacter* spp.; black stars—lymphoid follicles. Dog, H&E stain, Ob × 4 (**A**), Ob × 20 (**B**), Ob × 10 (**C**), Ob × 100 (**D**); scale bar = 500 µm (**A**), 100 µm (**B**), 200 µm (**C**), 10 µm (**D**).

All *H. pylori* infections start with acute gastritis which can persist ofr over 15 days after colonization. In a few cases healing moves into the chronic form. Tissue changes are characterized by degradation of the surface epithelium, the presence of numerous neutrophils located inside the epithelium, in the lamina propria, or aggregated in glandular lumens (cryptic abscesses). Acute gastritis induced by *H. pylori* is associated with nodular gastritis, follicular gastritis (Figure 2), hemorrhagic gastritis, lymphocytic gastritis, granulomatous gastritis, and hypertrophic gastritis. These lesions can be reversible [61,62].

**Figure 3:**
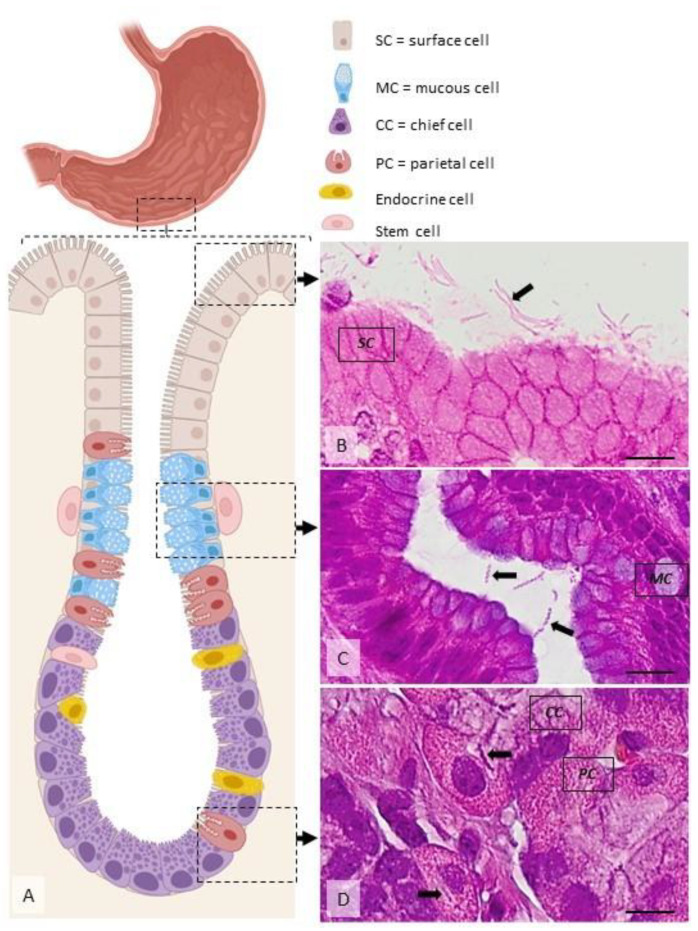
Distribution of *H. pylori* in the gastric mucosa. (**A**) Diagram showing the main cell types present within the gastric mucosa. *Helicobacter* spp. are located extracellularly, within the mucus covering the superficial mucosa ((**B**), arrow), within the glandular lumen ((**C**), arrows), or, rarely, intracellularly, in specific vacuoles ((**D**), arrows). Dog, H&E stain, Ob × 100; scale bar = 10 µm. Created using BioRender.

After the colonization of the gastric mucosa by *H. pylori*, the first disease produced by it is chronic active gastritis. Initially, chronic gastritis is superficial and limited to the antrum. As the infection persists, the lesions extend both in depth (atrophic gastritis) and on the surface of the gastric body (Figure 3). The persistence of injury to the gastric mucosa for a long time leads to the onset of hypo-/achlorhydria, maldigestion, malabsorption, and pernicious anemia [63]. The inflammatory process causes atrophy of mucosa and leads to intestinal metaplasia. The replacement of the gastric epithelium with the intestinal epithelium leads to the oncogenic risk increase and is proportional to the size of intestinal metaplasia areas. If the infection persists in time, in addition to the granulocyte infiltrate, lymphoid aggregates appear in the lamina propria of the mucosa, similar to lymphoid follicles (Peyer’s patches) in the small intestine (Figure 2). Gastric follicles become hyperplastic depending on the extension and duration of colonization of the stomach with *H. pylori* and are possibly at the origin of the development of a primary non-Hodgkin’s gastric lymphoma in the lymphoid tissue of the gastric mucosa [64].

Atrophic and metaplastic gastritis represents a chronic infection characterized histologically by a reduced number of epithelial cells and gastric glandular cells that evolve into foci or diffuse in gastric mucosa. It can be found as nodular gastritis, hemorrhagic gastritis, and hypertrophic gastritis [62].

Gastric ulcer is associated with antral gastritis in over 70% of cases. Erosions and gastric ulcers are the results of multifactorial action that alters the defensive barrier and the repair mechanisms of the gastric mucosa. All the factors that determine the increase in gastric acidity, the reduction of the recovery capacity of the epithelial cells predispose to the appearance of erosions and the formation of gastric ulcers [64]. The gastric mucosa is well-protected against bacterial infection, but *H. pylori* is well-adapted to this biological niche with unique characteristics, which allow it to enter the mucous layer, attach to epithelial cells, avoid the immune response, and, consequently, evoke persistent colonization and transmission. Mucosal colonization is due to a set of enzymes (urease, mucinase, peptidases, etc.) that provide it with pathogenicity, *H. pylori* being located under the mucous layer, between epithelial cells, and around the gastric crypts [65].

The emergence of gastric ulcers is due to two major pathogenic mechanisms: the direct one characterized by the decrease in mucosal resistance to aggressive factors where the inflammatory process initiated by *Helicobacter* toxins triggers acute gastritis that later becomes chronic and the indirect one characterized by stimulation of antral G cells, followed by increased gastric acid secretion and decreased secretion of somatostatin, all of which lead to an increased quantity of gastrin, especially postprandially [47].

### 3.2. Gastric Neoplasia Induced by H. pylori Infection

The gastric oncogenesis induced by *H. pylori* is complex and involves the sequential action of several pathogenicity factors (Table 1) and oncogenic mechanisms. There are several types of malignant gastric tumors induced by the chronic *H. pylori* infection, the most important in terms of both prevalence and aggressivity being gastric adenocarcinoma and gastric lymphoma.

Gastric adenocarcinoma. Gastric cancer is one of the most common types of tumors in the world. The fact that the major cause of gastric cancer is chronic *H. pylori* infection led to the classification of these bacteria in 1994 by the International Agency for Research on Cancer (IARC) under the World Health Organization as group I carcinogens, *H. pylori* being incriminated with certainty in the carcinogenesis of the gastric area. The recent studies of the experimental *H. pylori* infection of the Mongolian gerbil, an animal that is currently one of the model species for the study of *Helicobacter*-induced gastric tumors, have concluded that typical histopathological changes associated with this infection include chronic gastritis with subsequent metaplasia, intestinal and gastric mucosal atrophy. Most cases of gastric cancer develop into multifocal gastric atrophy, usually with extensive intestinal metaplasia, suggesting that metaplasia and gastric atrophy are precancerous lesions of the stomach [66,67]. *H. pylori* can produce tumor deviation of gastric cells in two ways: indirectly, by mutations of gastric epithelial cells as a collateral lesion of the inflammatory chain caused by the chronic presence of *H. pylori*, and directly, by the direct activity of the bacteria on the gastric epithelium or by the action of metabolites produced by the bacteria acting in the direction of alteration of the apoptosis/cell proliferation balance, impaired gene expression by action on transduction pathways, increased oxidative stress, alterations of intracellular toxic adhesion, direct action on epithelial cells, destruction of cellular DNA. Both bacterial proteins and cellular cytokines have a chemotactic role in inflammatory cells. Once activated, inflammatory cells produce chemical mediators represented by reactive oxygen species (ROS) while raising tissue levels of interleukin 8 (IL-8) [68].

Gastric lymphoma. The term “malignant lymphoma” suggests a more frequent malignant tumor of the lymph node tissue. In 1990, the association between *H. pylori* infection and gastric lymphoma was found; 92–97% of cases with gastric MALT lymphoma also showed lesions of chronic *Helicobacter* gastritis as well as the fact that antibiotic treatment to eradicate the infection may cause spontaneous remission of lymphoma in approximately 70% of patients, including treatment of gastric MALT lymphoma as a mandatory measure to eradicate *H. pylori* infection [69,70]. In humans, it has been established that in the case of *Helicobacter* infection, there is a 6.3% chance that dysplasia such as gastric MALT lymphoma will occur during life [67]. The mechanisms of action of *H. pylori* in the production of gastric MALT lymphoma are related to the proliferation of B lymphoma cells, proliferation stimulated by cytokines released by gastric T lymphocytes activated by the presence of *H. pylori* in the stomach [71]. MALT lymphoma occurs in normal lymphoid tissue but is preceded by the presence of chronic inflammation of lymphoid tissue, most commonly autoimmune. Paradoxically, although it does not normally have lymphoid tissue, the stomach is the most common site of extraganglionic lymphoma [72].

### 3.3. Extragastric Pathology Induced by H. pylori Infection

Although *H. pylori* infection is limited to the stomach, it triggers a significant systemic immune response in the patient. As a result, unfavorable effects of this reaction may lead to disease development in sites other than the gastrointestinal system.

Cardiovascular diseases. Lai et al. [73] have established that *H. pylori* infection significantly raises the risk of acute coronary syndrome. A meta-analysis of 26 studies including over 20,000 individuals performed by Liu et al. [71] found a significant link between *H. pylori* infection and the risk of myocardial infarction [74]. In patients with coronary artery disease (CAD), Lenzi et al. [75] investigated the overall prevalence of *H. pylori* and CagA-positive *H. pylori* infection, as well as the prevalence of other bacterial and viral causes of chronic infection, as well as the potential role of anti-heat shock protein 60 antibodies in increasing the risk of cardiovascular disease development [75]. *H. pylori* antibodies were found in the blood of patients with cardiovascular problems according to several seroepidemiologic studies. Atherosclerosis and chronic idiopathic thrombocytopenic purpura are two areas where *H. pylori* are likely to play a role, at least in a subset of individuals [76,77,78,79].

Hepatobiliary diseases. Hepatobiliary illnesses ranging from chronic cholecystitis and primary sclerosing cholangitis to gallbladder carcinoma and primary hepatic carcinomas have been linked to *Helicobacter* spp. that can invade the biliary tract. *H. pylori* have evolved adaptive methods to shield themselves from the stomach’s acidic environment. *Helicobacter* must have protection mechanisms against the negative effects of alkaline pH and bile acids if they are to survive in the biliary tract. Differential expression of virulence factors by different *Helicobacter* spp. is studied to see if it can help them survive in diverse habitats [80,81]. Bile acids are known to have inhibitory effects on the *H. pylori*. influence on adhesion and proliferation. Taurine-conjugated bile acids are better for *H. pylori* survival than glycine-conjugated bile acids. In an acidic environment, taurine-conjugated bile acids are more stable than glycine-conjugated bile acids, which can enhance the survivability of *H. pylori*. Although it is well-understood that bacteria play a role in the production of pigment stones, the particular mechanisms involved in stone formation are unknown. Interactions between *Helicobacter* species are also possible through the formation of hydrolyzing bile, nucleating proteins, chronic inflammation, and enzymes, microorganisms, or slime can act as a nidus for foreign bodies [82,83,84]. In a mouse model, Ki et al. [85] found that *H. pylori* increase hepatic fibrosis. Bacterial infection is thought to play a role in the production of cholesterol gallstones, and recent research has discovered the presence of *Helicobacter* spp. in the hepatobiliary system [86]. Helicobacter species DNA was discovered in the gallbladders of Chinese patients with gallstone illnesses, showing that *Helicobacter* infection may simply be a cofactor in the production of gallstones rather than a major contributor [87]. The presence of *Helicobacter* DNA was found to be associated with an increase in the proliferating cell nuclear antigen labeling index in the biliary epithelium, suggesting that *Helicobacter* spp. may play a role in the pathophysiology of hepatobiliary cancer by accelerating biliary cell kinetics [88].

Respiratory diseases. Dela Pena-Ponce et al. [89] investigated the influence of *H. pylori* and CagA-positive strains on the activation of inflammation in a model of pediatric airway epithelium. *H. pylori* selectively induced IL-8 production via the p38 MAP kinase, suggesting that it may have a protective impact on allergic asthma. In contrast to what has been reported in the pediatric population, Wang et al. recently demonstrated that *H. pylori* infection is related to an elevated risk of adult-onset asthma in both the general population and patients with comorbidities [90].

Neurologic diseases. *H. pylori* infection and CagA-positive strains are linked to vitamin B12 deficiency and peripheral neuropathy via the promotion of atrophic gastritis, according to Lee et al., and Yang et al. recently demonstrated that *H. pylori* infection and CagA-positive strains are linked to vitamin B12 deficiency and peripheral neuropathy via the promotion of atrophic gastritis [91,92]. Some researchers looked at *H. pylori*’s potential role in neurodegeneration by hypothesizing various pathogenic processes, including a possible effect on tau phosphorylation. Despite this, Zhou et al. [93] found no evidence of *H. pylori* modifying Thr205, Th3231, Ser396, and Ser404 sites in the hippocampus and cerebral cortex, which are typical Alzheimer’s disease markers.

Dermatologic diseases. *H. pylori* has been connected to several skin inflammations. Despite the lack of direct evidence of gastritis, Lewinska and Wnuk [94] hypothesized that *H. pylori* cytotoxins may increase stress-induced skin cell senescence. In a study of 75 vitiligo patients, researchers discovered a link between active vitiligo and *H. pylori* infection. Eleven of the 49 participants indicated that their dermatologic signs had disappeared three months after receiving *H. pylori* eradication medication [95,96].

## 4. Alternative and Complementary AgNPs-Based Approaches in the Treatment of *Helicobacter pylori* Infection

Currently, due to the lack of an efficient strategy for eradicating *H. pylori* infection in the context of drug resistance, one of the focuses of research for a better therapeutic approach is on alternative and complementary methods, including AgNPs per se [97], AgNPs embedded in polymeric nanocarriers [98], or AgNPs combination with classical antibiotics [99].

### 4.1. Therapies Using AgNPs per se in the Eradication of Helicobacter pylori Infections

The persistence of gastric infections caused by *H. pylori* and the current failure of several therapies using antibiotics is based largely on the emergence of novel drug-resistant strains of *H. pylori.* This acquired resistance is an important challenge for pharmaceutical and biomedical research, forcing the scientific community to continuously develop new drug substances [100]. Resistance to antimicrobial agents may include changes in the permeability of the bacterial wall, elimination of antimicrobial agents by efflux of membrane pumps, changes in the sites of action of drugs, and inactivation of antimicrobial agents. Therefore, combating multiresistant pathogens with current medication is ineffective [101]. Development of nanoparticles in this field makes them an attractive alternative to antibiotics, having a high potential for resolving antibiotic resistance [102]. In addition, microorganisms are unlikely to develop resistance to the use of nanoparticles because nanoparticles attack different targets in the bacterium. This means that bacteria need to simultaneously develop a set of mutations to protect them, which is unlikely [103]. Recently, Haibo Wang et al. demonstrated that silver was efficient in inhibiting a key target 6-phosphogluconate dehydrogenase through binding to catalytic His185 by X-ray crystallography in *Staphylococcus aureus*. Furthermore, they managed to separate 38 authentic Ag+-binding proteins in *S. aureus* [104]. The same authors delineated the first antimicrobial action of Ag+ in *E. coli*. The principal effect was represented by damage of bacterial enzymes in glycolysis and tricarboxylic acid cycle determining the stalling of the oxidative branch of the tricarboxylic acid cycle. Furthermore, an article published in 2019 confirmed that silver ions target glyceradldehyde-3-phosphate dehydrogenase in glycolysis in *E. coli* [105,106]. The potential action of silver ions against bacteria should be taken into consideration as a better antimicrobial alternative treatment reducing the risk of antibiotic resistance.

Different types of nanoparticles have various mechanisms for microbial control. Most types of nanoparticles manage to overcome at least one of the common mechanisms of bacterial resistance. This effect is due to the bactericidal activity of nanoparticles based on specific physicochemical properties [107].

Currently, nanoparticles are considered an alternative to antibiotics, having an enormous potential to solve the problem of bacterial resistance to most antibiotics. Nanoparticles, especially silver nanoparticles, have attracted scientific attention. Potent antibacterial and broad-spectrum activity against bacteria with different morphology and metabolism is exemplified by the action against cholera, with the multifunctional mechanism of nanoparticles for interaction with each type of bacterium [108]. Silver nanoparticles can interact with the cell surface of various bacteria. This aspect is particularly important in the case of Gram-negative bacteria, in which case the adhesion and accumulation of silver nanoparticles on the bacterial surface have been observed in numerous studies [109]. Silver has been described as “oligodynamic” due to its antibacterial effect at very low concentrations. Silver ions are known to be highly reactive and can easily bind to a variety of negatively charged molecules, including inorganic anions, proteins, RNA, and DNA. Therefore, antibacterial properties have been attributed to its interaction with thiol but also with such groups as carboxylates, phosphates, hydroxyls, imidazoles, indoles, and amines [110].

Numerous studies have shown that silver nanoparticles are capable of damaging the cell membrane which can lead to structural changes, increasing the permeability of the bacterium. This aspect is influenced by the concentration, shape, and size of silver nanoparticles. A study that used *Escherichia coli* confirmed that the accumulation of silver nanoparticles on the surface of the cell membrane creates a gap in the integrity of the lipid bilayer which predisposes the cell to increased permeability, eventually leading to cell death [109]. Different studies have shown that the activity of silver nanoparticles is closely dependent on their size [111]. Small nanoparticles have a superior ability to penetrate bacteria. The interaction with the bacterial membrane and the resulting lesions leading to cell death are certainly much more evident in the case of small nanoparticles with positive zeta potential. The electrostatic force that occurs when nanoparticles with positive zeta potential encounter a bacterium whose surface is negatively charged leads to a very high attraction and inertia between the two entities and the possible penetration of nanoparticles into the cell membrane. The zeta potential together with the nanoparticle size is indeed a fundamental parameter in the control of antibacterial activity, the most effective being nanoparticles with small dimensions and positive zeta potential. Nanoparticles have a much higher antibacterial activity than silver-free ions; for this reason, the antibacterial activity is attributed to the physical properties of both nanoparticles and free ions. The combined effect of silver-free ions and nanoparticles produces a very strong antibacterial activity with a very broad spectrum [112]. Silver nanoparticles also damage the membrane by inducing the release of reactive oxygen species (ROS) to form free radicals that have strong antibacterial activity. Silver ions or small silver nanoparticles can easily penetrate cells, causing damage to intracellular structures [113].

### 4.2. Therapies Using AgNPs in Combination with Antibiotics or Embedded in Polymeric Nanocarriers in the Eradication of Helicobacter pylori Infections

A study by Mansouri et al. demonstrates that a combination of clarithromycin and AgNPs can suppress bacteria more effectively. This study showed that using AgNPs and clarithromycin together could reduce the MIC of certain *H. pylori* isolates by up to two times. A novel method for treating *H. pylori* infection uses this combination. It has already been proven that combining metallic NPs and antibiotics has a synergistic antibacterial effect against other bacterial strains. Today, it is indeed challenging to treat microbial infections with a single medication, but by combining different bactericidal mechanisms, the therapeutic efficacy can be increased [97]. Nanocarriers could be designed to distribute pharmaceuticals preferentially to specific places, shield drugs from hostile environments (such as the stomach), and improve cellular internalization to treat intracellular infections. Antimicrobial medicines against *H. pylori* are delivered in a variety of ways thanks to the use of nanocarriers. Polymer nanoparticles were used as nanocarriers for silver in a study by Camargo et al. They demonstrated an increased antibacterial activity of silver-based compounds enclosed into polymer nanoparticles compared with silver-based compounds alone [98].

## 5. The Antibacterial Mechanism of Silver Nanoparticles: An Extended Paradigm towards the Treatment of *Helicobacter pylori*

**Figure 4:**
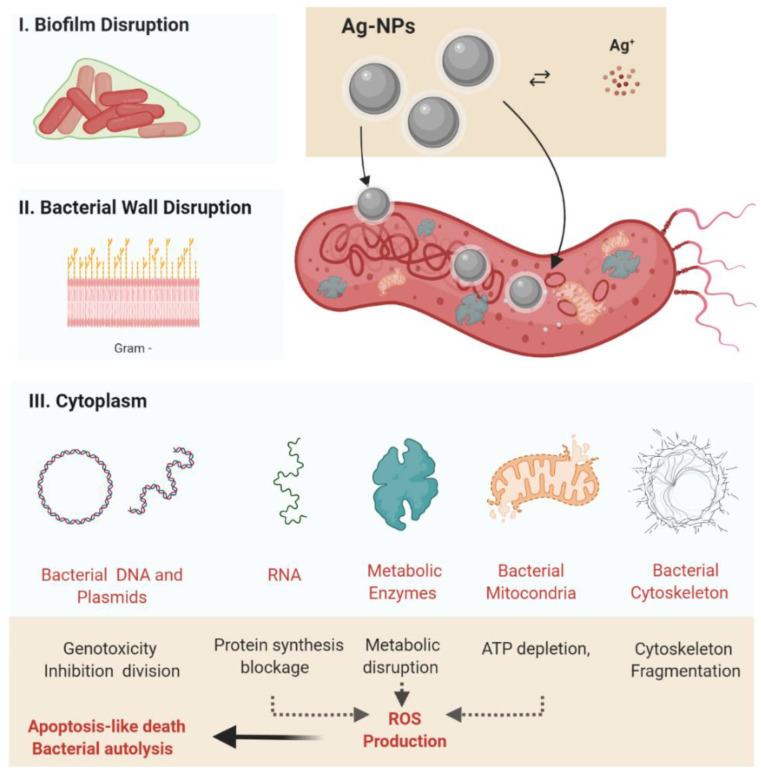
Silver nanoparticles’ antibacterial activity, highlighting the main action mechanisms (**I**). Disruption of the bacterial biofilm (**II**). Adherence of AgNPs to the bacterial cell wall, with bacterial membrane disruption followed by bacterial cytoplasm leakage and electrolytes transport disbalance (**III**). Direct interaction of the AgNPs present within the cytosol with bacterial organelles (mitochondria) and cytoplasmic molecules (including enzymes and the cytoskeleton) followed by metabolic disruption. Abbreviations: AgNPs, silver nanoparticle; ROS, reactive oxygen species: Ag+, silver ions. Created using BioRender (adapted from [117]).

Silver nanoparticles have a complex antibacterial activity involving a synergistic mechanism mediated by direct disruption of the bacterial cell wall, alteration of bacterial metabolism, and oxidative stress by the generation of ROS (Figure 4) [97,113,114] In addition, the ability of nanoparticles to penetrate the bacterial biofilm also provides us with a practical method to inhibit biofilm formation. The nanoparticles must come into contact with the bacterial cell to exert their antibacterial function. Accepted forms of contact include electrostatic attraction, van der Walls forces, and receptor–ligand and hydrophobic interactions. The interaction of nanoparticles with the basic cellular components of the bacterium (DNA, lysozymes, ribosomes, and enzymes) leads to oxidative stress, heterogeneous alterations, changes in cell permeability, electrolyte imbalances, enzyme inhibition, protein deactivation, and changes in gene expression [115].

The current paradigm on the antibacterial effect of silver nanoparticles indicates (1) the oxidative stress (mainly following generation of ROS), (2) the release of metal ions, and (3) non-oxidative processes as the main mechanisms explaining the antibacterial properties [114,115]. While nonoxidative processes involve mainly a direct mechanical action of AgNPs on the bacterial wall and internal structures, the oxidative stress and the generation of metal ions is more complex and will be briefly explained further.

### 5.1. Oxidative Stress

Reactive oxygen species (ROS) induced by oxidative stress are an important antibacterial mechanism of nanoparticles. ROS is a generic term for molecules and reactive intermediates that have a strong redox potential, and different types of nanoparticles produce different types of ROS by reducing oxygen molecules. The four reactive oxygen species are superoxide radical (O-2), hydroxyl radical (•OH), hydrogen peroxide (H_2_O_2_), and single oxygen (O_2_). Typically, the production and release of reactive oxygen species into bacterial cells are balanced. If there is an excessive increase in ROS production, the cellular redox balance favors oxidation. This state of imbalance produces oxidative stress that leads to the damage and destruction of bacterial cell components [116]. Oxidative stress has been confirmed to have a key contribution to changes in cell membrane permeability, which can lead to the destruction of the bacterial membrane [117]. It has been confirmed that Al_2_O_3_ nanoparticles cross the cell membrane reaching intracellular structures, and the interaction of nanoparticles with the membrane eventually leads to the loss of its integrity, most likely due to intracellular oxidative stress. Zinc oxide nanoparticles can break down H_2_O bonds in H+, and in an environment containing water and oxygen, they can react with dissolved oxygen to generate H_2_O_2_. These reactive oxygen secretions can penetrate the cell membrane, destroying the bacteria [114].

### 5.2. Release of Metal Ions

Metal ions are slowly released from metal oxides and are absorbed through the cell membrane following a direct interaction with functional protein groups and nucleic acids such as thiol amino (-NH), and carboxyl groups (-COOH), modifying the cellular structure, affecting the enzymatic activity and normal physiological processes, eventually reaching the inhibition of the microorganism. However, the impact of metal ions on the pH inside the lipid vesicles is reduced during the antibacterial process of metal oxide suspension, having a weak antimicrobial activity [118]. Therefore, dissolved metal ions are not the main antibacterial mechanism of metal oxide nanoparticles [119]. Similar studies have shown that supermagnetic iron oxide interacts with microbial cells by directly penetrating the cell membrane and interfering with transmembrane electron transfer. In addition, metal ions can act as carriers of antimicrobials [114].

### 5.3. Stages of Antibacterial Activity of Silver Nanoparticles

The above-described antibacterial mechanisms, although synergic, have a sequential action on the bacterial structure, involving a step-by-step interaction with extracellular polymers, bacterial cell wall, cytosolic enzymes, bacterial DNA, and organelles. In the next section, the main steps involved in the antibacterial effect are detailed.

#### 5.3.1. The Interaction of Nanoparticles with the Bacterial Cell Wall

The cell wall and membranes are important defensive barriers for bacterial resistance to external environmental factors. The cell wall plays an important role in maintaining the natural shape of the bacterium. The components of the cell membrane of Gram-positive and Gram-negative bacteria produce different adsorption pathways for nanoparticles [120]. LPS is a unique cell wall structure of Gram-negative bacteria that provides a negative surface charge in certain regions by attracting nanoparticles. Instead, teichoic acid is present only in the cell wall of Gram-positive bacteria, the nanoparticles being distributed over the entire surface of phosphate molecular chains preventing aggregation. Numerous studies have suggested that nanoparticles have a higher activity against Gram-positive bacteria than against the Gram-negative ones because the cell wall of negative bacteria is composed of LPS, lipoproteins, and phospholipids, which form a penetrable barrier only for macromolecules. Instead, the wall of Gram-positive bacteria contains a thin layer of peptidoglycans, teichoic acids, and numerous pores that allow foreign molecules to enter, resulting in membrane damage and ultimately cell death. In addition, compared to Gram-negative bacteria, the Gram-positive ones have a negative surface charge, which increases the nanoparticles [121]. The mechanism by which nanoparticles cause cell death is dependent on the structure and components of the bacterial cell. For example, the antimicrobial action of ZnO is dependent on the specific composition of the bacterial cell, which can be improved for Gram-positive bacteria. Some special components of Gram-negative bacteria such as LPS can prevent the adhesion of ZnO nanoparticles to the cell barrier by regulating the flow of ions entering and leaving the cell membrane. The wall thickness of Gram-negative bacteria affects the activity of nanoparticles [107].

Foster et al. [122] confirmed that titanium dioxide nanoparticles can adhere to the bacterial cell surface producing reactive oxygen species by damaging cell membrane components and structure by interfering with cell membrane function, causing cell contents to leak, leading to cell death. Iron can cause cellular decomposition, and nanoparticles can cause cellular aggregation that leads to loss of function through cellular compression. Physical destruction of the cell membrane is one of the most important mechanisms of antibacterial activity [122].

#### 5.3.2. Inhibition of Bacterial Protein and DNA Synthesis

Su et al. [123] studied the influence of CuO nanoparticles on bacterial denitrification causing a significant alteration of key protein expression. After entering the cell, CuO nanoparticles cause the regulation of proteins involved in nitrogen metabolism, electron transfer, and substance transport [123]. The antibacterial mechanism of silver nanoparticles is closely related to the depletion of antioxidant capacity. Fucoidan proteins, which are critical in maintaining cell morphology and directing the movement of iron and other chemicals across the membrane, have been demonstrated to be attacked by nanoparticles, resulting in cell membrane discontinuities [114].

### 5.4. In Vitro Experiments Assessing the Anti-H. pylori Activity of Silver Nanoparticles

Several studies regarding the efficiency of silver nanoparticles in the treatment of *H. pylori* infection have been published in the last decade, and they are listed in Table 2. Fifteen important studies were performed in vitro using several extract types of silver nanoparticles and silver ultrananoclusters on different strains of *H. pylori* bacterial cultures. Non-spherical silver ultrananoclusters, with the smallest size of all the AgNPs, were used in a single study [124], and they showed a high degree of growth inhibition of the bacterium when the dosage was between 0.16 and 0.33 mg/L. In the vast majority of the studies, spherical silver nanoparticles were used, obtained from different extracts, having various sizes: *A. priceps*, 5–30 nm [125]; *S. xanthocarpum*, 4–18 nm [32,126]; *T. vernicifluum*, 4–20 nm [127]; *C. longa* [128] and *A. nilotica*, 22–66 nm [124]; *P. putida*, 6–16 nm [35]; *A. calamus*, 5–60 nm [129]; *A. nilotica*, 22–55 nm [130]. The most common effect on the bacterial colonies was inhibition of growth, which was presented in six of the papers [124,125,127,128,131,132,133,134], followed by activation of reactive oxygen species production [130,132], inactivation of *H. pylori* urease [131,135], bacterial DNA fragmentation and consecutive loss of viability [130], damage to the bacterial cellular membrane [132], and activation of antioxidant activity [136].

One study was performed using a silver compound loaded into polymeric nanoparticles with a good minimum inhibitory concentration (3.90 μg/mL) [98].

### 5.5. In Vivo Experiments Assessing the Anti-H. pylori Activity of Silver Nanoparticles

There are few in vivo studies addressing the antibacterial effect of AgNPs against *H. pylori* (summarized in Table 3). One relevant study was performed in vivo by Kuo et al. [136] on a batch of 8-week-old gerbils with bodyweight between 30 and 40 g which were fed with AgNp/clay complex at 0.1% of their bodyweight and another batch that was fed with the same complex at 1% of their bodyweight. In a study in 2020, Al-Bahrani et al. showed a good inhibition of growth using an extract of *A. bisporus* [138]. Another study published in the same year carried out by Amin et al. [131] was performed on 72–112-day-old male albino Wistar rats weighing around 295 g. Ag nanoparticles from *P. harmala* seeds were used in a dosage between 1 and 32 μg/mL. The results showed a small degree of growth inhibition of the bacterium compared with the studies conducted in vitro where the AgNP/clay complex showed a more obvious inhibitory effect. A study conducted in 2022 demonstrated a good efficacy of AgNPs in combination with clarithromycin compared with clarithromycin treatment only [99].

## 6. Other Metal-Based Nanoparticles Used in the Treatment of *Helicobacter pylori* Infection

The conventional therapy for *H. pylori* infection already includes medications containing bismuth. Since the early 19th century, they have been used to treat digestive complaints; nonetheless, their antibacterial action remains poorly known, and no cases of bismuth resistance have been reported [97,140,141]. An extended analysis of 41 studies by Catherine Gomez et al. showed an important antibacterial effect of bismuth-based nanoparticles [135]. Griffith et al. provided important information regarding the unique effect of bismuth against *H. pylori* infection [142]. Another study, published in 2006, showed that bismuth subcarbonate nanotubes demonstrated antibacterial activity against the bacterium *H. pylori*, which causes gastritis and peptic ulcers. For the treatment of *H. pylori* infection and possibly other disorders, research is currently conducted to insert additional medications into nanotubes to create “nanodrugs” [143]

Chakraborti et al. [144] proved that polyethyleneimine-functionalized zinc oxide nanoparticles (ZnO-PEI NPs) are readily internalized in *H. pylori*, causing cell membrane disruption, RNA degradation, morphological transformation into spheroids, and, in some cases, loss of vitality.

Gopinath et al. [35] demonstrated that sized gold nanoparticles (GNPs) have anti- *H. pylori* efficacy against multidrug-resistant *H. pylori* strains in a study using the dried fruit extract of *Tribulus terrestris*. Furthermore, biogenic GNPs have good catalytic activity for converting poisonous p-nitroaniline to a nontoxic byproduct, p-phenylenediamine. Wu et al. [145] showed in vitro that an ultralow concentration of Mn_0.3_Fe_2.7_O_4_@SiO_2_ nanoparticles exposed to a moderate AC magnetic field may deposit heat locally and efficiently limit *H. pylori* growth and virulence without causing bulk heating. In comparison to amoxicillin treatment and nanoparticle heating alone, dual-functional amoxicillin-loaded Mn_0.3_Fe_2.7_O_4_@SiO_2_ further reduces bacterial viability by a factor of 7 and 5, respectively, when combined with antibiotic amoxicillin. The synergistic effect may be due to heating-induced disruption of the cell membrane and protective biofilm, which could increase antibiotic permeability of bacteria [145].

A study conducted by Umamaheshwari and Jain [146] looks at the possibility of employing lectin-conjugated gliadin nanoparticles to locate and anchor a drug delivery system on *H. pylori* carbohydrate receptors. A desolvation process was used to make gliadin nanoparticles (GNP) containing acetohydroxamic acid (AHA). The two-stage carbodiimide coupling approach was used to bind the lectins *Ulex europaeus* agglutinin I (UEA I) and conconavalin A (Con A) to GNP formulations. The binding effectiveness of lectin formulations to carbohydrate receptors of *H. pylori* strains was evaluated using a lectin agglutination assay. When compared to GNPs, the inhibitory efficacy of UEA–GNPs and Con A–GNPs was roughly two-fold higher. These lectin-conjugated gliadin nanoparticles have been discovered to be a promising candidate for targeted medication administration and could be effective in the treatment of *H. pylori* [146]. Chiung-Hung et al. used berberine, a plant alkaloid, that has been shown to drastically inhibit *H. pylori* multiplication [147]. This work produced a new nanoparticle berberine carrier with a heparin shell to localize berberine to the location of *H. pylori* infection. The suggested in vitro drug carrier system efficiently controlled the release of berberine, which interacted selectively with the intercellular space at the site of *H. pylori* infection, according to an analysis of a simulated gastrointestinal medium. Furthermore, the nanoparticles effectively reduced cytotoxic effects in *H. pylori*-infected cells while considerably increasing the suppressive effect of berberine on *H. pylori* growth [147].

## 7. Conclusions

Due to the high prevalence and emergence of antibiotic-resistant strains, *H. pylori* represents a major global public health problem, but currently, nanoparticle research can offer novel, exciting therapeutic alternatives which may prove to be valuable tools in improving *H. pylori* treatment and infection prevention. The current manuscript provides an overview of the pathogenesis of *H. pylori*-induced gastric and extradigestive diseases and highlights the possible benefits and limitations of metallic nanoparticles in the therapy and prevention of *H. pylori* infections. Despite promising in vitro studies, as of today, there have been only few in vivo studies assessing the potential usage of AgNPs against *H. pylori* infections. This limits the translational value of these promising results, urging the need for experimental usage of more complex *H. pylori*-induced infection models.

## Figures and Tables

**Table 2:** Synopsis of the in vitro studies using AgNPs in the treatment of *Helicobacter* spp.-induced diseases.

	*Helicobacter* Species/Strain	Experimental Model	Nanoformulation	Tested Doses	NP Shape and Size Distribution	Effect on Bacteria	References
1.	*H. pylori* (NCTC 11638 strains)	Bacterial culture	AgNPs (extract of *Solanum xanthocarpum)*	2 μg/mL^−1^ MIC	Spherical 4–18 nm	Inactivation of *H. pylori* urease	[32]
2.	*H. pylori* (clinical isolates from humans)	Bacterial culture	AgNPs (extract of *Solanum xanthocarpum)*	2–8 μg/mL^−1^ MIC	Spherical 4–18 nm	Inactivation of *H. pylori* urease	[126]
3.	*H. pylori* I (NCTC 11637 strains)	Bacterial culture	AgNPs (extract of *Solanum xanthocarpum)*	4 μg/mL^−1^ MIC	Spherical 4–18 nm	Inactivation of *H. pylori* urease	[126]
4.	*H. pylori* (NCTC 11637 NCTC 11638)	Bacterial culture	AgNPs (*P. harmala* seeds)	4.0 μg/mL (NCTC 11637) 8.0 μg/mL (NCTC 11638) MIC	Spherical	Inhibition of growth	[131]
5.	*H. pylori* (GS-13 Strain)	Bacterial culture	AgNPs (leaf extract of *A. priceps)*	5.0 μg/mL MIC	Spherical 5 ± 30 nm (average, 20 nm)	Inhibition of growth Loss of viability ROS production DNA fragmentation	[125]
6.	*Helicobacter felis* (GS-14 strain)	Bacterial culture	AgNPs (leaf extract of *A. priceps)*	5.5 μg/mL MIC	Spherical 5 ± 30 nm (average, 20 nm)	Inhibition of growth Loss of viability ROS production DNA fragmentation	[125]
7.	*H. pylori* (UM066), *H. pylori* J99, and *H. pylori* NCTC 11637	Bacterial clinical isolates	AgNPs (soil-derived *Pseudomonas putida* MVP2)	24 μg/mL	Spherical 6–16 nm	Inhibition of growth	[132]
8.	*H. pylori* (MH179988)	Bacterial culture	Tv-AgNPs (extract of *Toxicodendron vernicifluum*)	18.14 μg/mL^−1^ MIC	Spherical 2–40 nm	Inhibition of growth Damage to the bacterial membrane ROS production	[127]
9.	*H. pylori* (BHI)	Bacterial culture	AgNPs (extract of *Curcuma longa)*	N/A dosage data	Spherical No size data	Inhibition of growth	[128]
10.	*H. pylori* biofilm	Bacterial culture	AgNPs (extract of *Acorus calamus* L.)	350 μg/mL (highest activity)	Spherical and near-spherical 5 to 60 nm	Inhibition of growth	[129]
11.	*H. pylori* (ATCC 26695).	Bacterial culture	py-AgNPs (*Acacia nilotica* leaf extract-mediated compound pyrogallol)	10–40 μg/mL	Spherical 22.68–55.16 nm	Antioxidant activity	[130]
12.	*H. pylori* (ATCC 43504)	Bacterial culture	Silver ultrananoclusters	0.16 to 0.33 mg/L	Non-spherical shape 1.83 ± 1.57 nm	Inhibition of growth	[124]
13.	*H. pylori* ATCC 43504	Bacterial culture	AiiA-AgNPs	1–5 μM	No shape and size data	Inhibition of growth	[133]
14.	Urease (from jack beans)	Bacterial culture	AgNPs (leaf extract of *Ficus carica*)	N/A dosage data	Spherical 21 nm (average size)	Urease inhibition	[137]
15.	*H. pylori* strain 43504	Bacterial culture	Ag (PhTSC∙HCl)_2_ (BPN: base polymeric nanoparticle)	3.90 μg/mL MIC	Spherical shape No size data	Inhibition of growth	[97]
16.	*Helicobacter pylori* (ATCC 43504^TM^)	Bacterial culture	LEVB-AgNAPs (leaf extract of *Viola betonicifolia*)	120 μg/mL	6–11 nm No shape data	Inhibition of growth	[134]

MIC—minimum inhibitory concentrations.

**Table 3:** Synopsis of the in vivo studies using AgNPs in the treatment of *Helicobacter* spp.-induced diseases.

	*Helicobacter* Species/Strain	Experimental Model	Nanoformulation	Tested Doses	NP Shape and Size Distribution	Effect on Bacteria	References
1	*H. pylori* (NCTC 11637)	Male albino Wistar rats (72–112 days and weight of 295 ± 4.1 g)	AgNPs (*P. harmala* seeds)	1–32 μg/mL MIC	Spherical 15–18 nm	Inhibition of growth	[131]
2	*H. pylori* (metronidazole-resistanr strain)	50 biopsy samples	Ag_2_ONPs (*Digiria muricata*)	25–100 μg/mL	11–35.6 nm	Inhibition of growth	[139]
3	*H. pylori* (no strain data)	Eight-week-old gerbils with a bodyweight of 30–40 gm	AgNPs (lucentite SWN clay slurry)	0.1% weight	No shape and size data	Inhibition of growth	[136]
4.	*H. pylori* (no strain data)	50 biopsies from patients with duodenal ulcer	AB-AgNPs (extract of *A. bisporus*)	25–100 mg/mL	No shape and size data	Inhibition of growth	[138]
5.	*H. pylori* (no strain data)	40 gastric biopsies	AgNPs	31.25–250 μg/mL MIC	Sspherical 5–8 nm	Inhibition of growth	[99]
6.	*H. pylori* (no strain data)	40 gastric biopsies	AgNPs in combination with clarithromycin	31.25–125 µg/m MIC	Spherical 5–8 nm	Inhibition of growth	[99]

## Data Availability

Not applicable.

## Supplementary Materials

The following supporting information can be downloaded at: https://www.mdpi.com/article/10.3390/pharmaceutics14071463/s1. File S1. “PRISMA Diagram”.
