# Supplementary material for: Helicobacter Pylori-Induced Gastric Infections: From Pathogenesis to Novel Therapeutic Approaches Using Silver Nanoparticles
Source: Pharmaceutics. 2022 Jul 14;14(7):1463. doi: 10.3390/pharmaceutics14071463 (PMC9318142; doi:10.3390/pharmaceutics14071463)
Supplement: Supplementary file 1 [file pharmaceutics-14-01463-s001.zip › pharmaceutics-1777085-supplementary.pdf]

File S1

PRISMA 2020 flow diagram for new systematic reviews which included searches of databases only

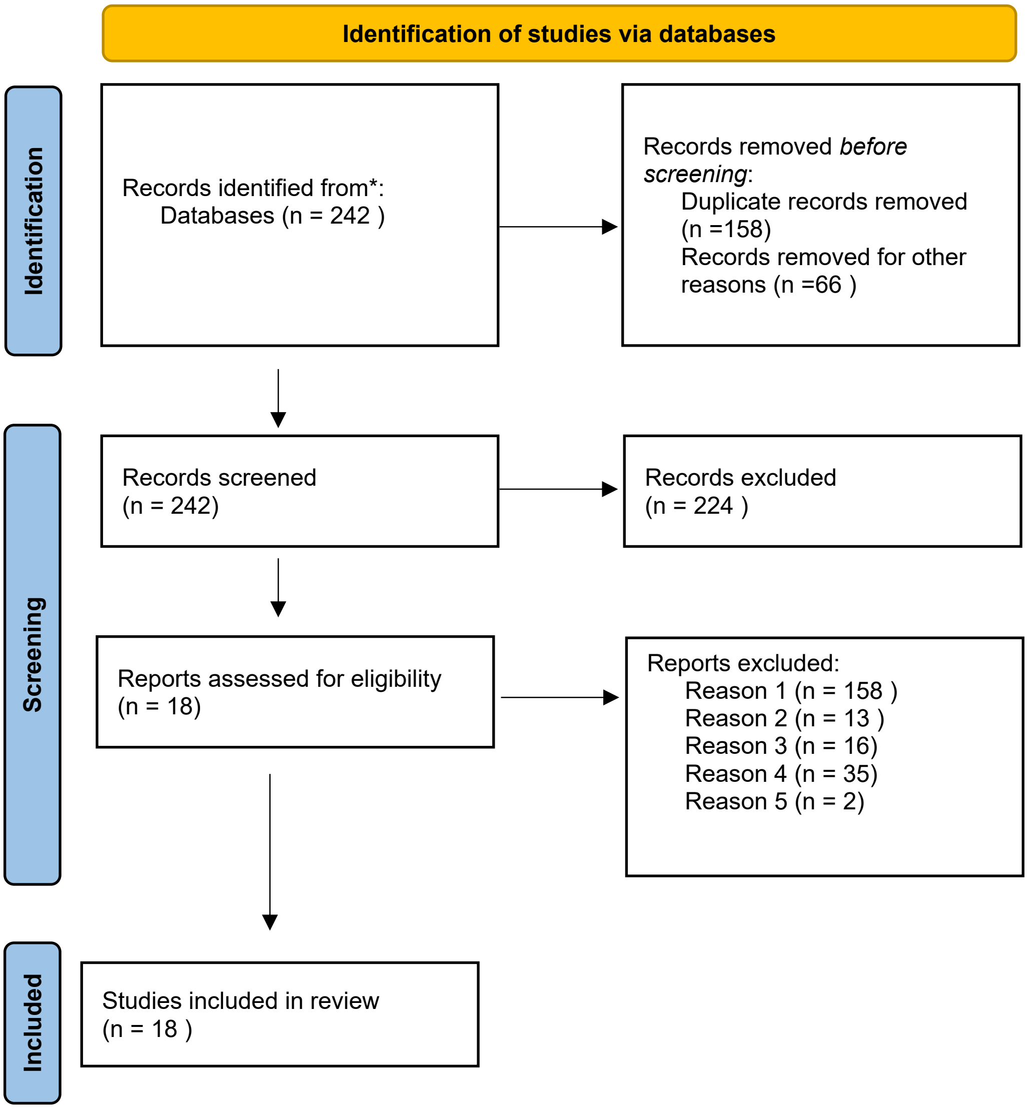

Reason 1 – duplicate

Reason 2- "Helicobacter pylori" is not found in the content

Reason 3-"Silver nanoparticles" not found in the content

Reason 4- both "Helicobacter pylori" and "Silver nanoparticles" were not found in the content

Reason 5- were review articles
